# Association of glycemic variability and sociodemographic determinants with periodontal health in children with type 1 diabetes: a cross-sectional study from Türkiye

**DOI:** 10.1186/s12903-025-07484-z

**Published:** 2026-02-28

**Authors:** Gülay Can Yılmaz, Özgül Cartı Dörterler

**Affiliations:** 1 Department of Pediatric Endocrinology, Faculty of Medicine, Muğla Sıtkı Koçman University, Aydın Muğla Yolu No:50, Muğla Merkez/Muğla, 48000 Türkiye; 2 Department of Pediatric Dentistry, Faculty of Dentistry, Muğla Sıtkı Koçman University, Orhaniye Mahallesi Haluk Özsoy Caddesi No:4 P.K, Menteşe/MUĞLA, 48000 Türkiye

**Keywords:** Type 1 diabetes mellitus, Pediatric dentistry, Periodontal health, Xerostomia, Glycemic control, Glycemic variability, Plaque index, Gingival index

## Abstract

**Background:**

Periodontal complications are frequently reported in youth with type 1 diabetes (T1DM), yet evidence linking continuous glucose monitoring (CGM) metrics to pediatric periodontal status is limited. We assessed periodontal health in T1DM versus healthy peers and examined associations of HbA1c and CGM-derived metrics—including coefficient of variation (CV), time in range (TIR), and time below range (TBR)—with periodontal indices, alongside sociodemographic determinants.

**Methods:**

In this single-center cross-sectional study (age 7–12 years), T1DM participants (*n* = 52) were compared with healthy controls (*n* = 26). Periodontal outcomes were Plaque Index (PI), Gingival Index (GI), and simplified Basic Periodontal Examination (s-BPE). CGM summaries (preceding 14–30 days) were obtained for T1DM only. Caries experience (dmft/DMFT) was recorded as an oral-health descriptor. Group comparisons used Kruskal–Wallis/Dunn tests; associations used Spearman correlations and multivariable linear regression adjusting for age, sex, parental ages, education, and household income.

**Results:**

Compared with controls, T1DM showed higher PI, GI, and s-BPE (all *p* < 0.001). Xerostomia was more frequent in T1DM (30.8% vs. 0%; *p* = 0.004). Periodontal indices did not differ between HbA1c (< 7% vs. ≥ 7%) or CV (< 36% vs. ≥ 36%) strata. Within T1DM, TBR < 70 mg/dL correlated weakly but positively with PI (ρ = 0.312; *p* = 0.024) and GI (ρ = 0.316; *p* = 0.022). In adjusted analyses restricted to the T1DM cohort, time below range < 54 mg/dL (TBR < 54) was positively associated with the plaque index. For the gingival index, small associations were observed with time in range (TIR) and glycemic variability (CV); however, these were directionally inconsistent with biological expectations and should be interpreted cautiously. No predictors were identified for the s-BPE screening outcome. dmft/DMFT did not differ across groups; maternal education associated with lower DMFT (*p* < 0.05). Rural–urban residence showed no differences.

**Conclusions:**

Children with T1DM exhibit poorer periodontal health than healthy peers. An exploratory link between higher hypoglycemia burden (TBR) and greater plaque/gingival inflammation suggests a modifiable behavioral conduit—hypoglycemia management and night-time hygiene—superimposed on salivary dysfunction and social determinants. Findings support integrating CGM-informed counseling on free-sugar exposure and oral hygiene into pediatric diabetes care and align with SDG-3 goals to strengthen prevention within routine services.

**Supplementary Information:**

The online version contains supplementary material available at 10.1186/s12903-025-07484-z.

## Introduction

Type 1 diabetes mellitus (T1DM), the most common form of pediatric diabetes in many regions, results from autoimmune destruction of pancreatic β-cells [1]. In 2021, an estimated 537 million adults worldwide had diabetes, and more than 1.2 million children and adolescents < 20 years were living with T1DM [2].

Diabetes is a systemic disease with well-recognized oral manifestations. Pediatric studies frequently report greater plaque accumulation and gingival inflammation in T1DM than in healthy peers, particularly among those with poorer metabolic control [3–5]. Salivary dysfunction—reduced flow and/or altered composition—and xerostomia are also implicated, with potential consequences for biofilm and gingival health [5, 6]. However, while a diabetes–periodontitis link is widely discussed, pediatric evidence remains heterogeneous [7–9].

Whether metabolic control per se explains periodontal status remains uncertain. Some pediatric studies link higher HbA1c to greater periodontal inflammation/plaque [10, 11], whereas others report partial or null associations [12]. Because HbA1c reflects mean glycemia rather than short-term fluctuations or hypoglycemia exposure, continuous glucose monitoring (CGM) provides complementary characterization. Consensus recommends reporting coefficient of variation (CV), time in range (TIR; 70–180 mg/dL), time below range (TBR < 70 mg/dL, including time < 54 mg/dL), and time above range (TAR; >180 and > 250 mg/dL), with pediatric targets such as CV < 36% and TIR ≥ 70% [13, 14]. TIR correlates strongly with HbA1c and is increasingly used clinically; notably, higher TIR is associated with fewer microvascular complications in adults independent of HbA1c, and in youth, real-time CGM—particularly with pump therapy—supports achieving TIR ≥ 70% and reduces severe adverse events [15–17]. Pediatric studies examining periodontal outcomes in T1DM have largely relied on HbA1c rather than standardized CGM metrics [18, 19], despite consensus recommending CV, TIR, TBR, and TAR [13, 14]. To our knowledge, few studies have related these CGM-derived metrics to pediatric periodontal indices while adjusting for key behavioral and sociodemographic factors.

Beyond metabolic control, social and behavioral determinants—including daily oral-hygiene practices, caregiver education/health literacy, socioeconomic conditions, and access to dental care—also influence pediatric oral outcomes, though their relative contributions in T1DM remain uncertain [20, 21]. Consistent with this, caregiver oral-health literacy and family-level characteristics have been linked to children’s home-care behaviors and clinical oral-health status [21, 22]. In line with this broader context, pediatric T1DM cohorts show heterogeneous findings—e.g., higher plaque/gingival bleeding in some cohorts, whereas others report poor oral health irrespective of HbA1c, with behaviors (tooth-brushing/flossing) emerging as key correlates [18, 19].

Dietary free-sugar exposure is another important determinant of pediatric oral health and may interact with glycemic status [23, 24]. In adults, limited randomized evidence suggests that anti-inflammatory or Mediterranean-style dietary patterns can reduce gingival inflammation even without changes in plaque control [25–27]. In youth with T1DM, treatment of impending hypoglycemia with rapid-acting carbohydrates can increase free-sugar exposure [28].

Accordingly, this comparative cross-sectional study primarily aimed to examine associations of HbA1c and CGM-derived glycemic metrics (CV, TIR, TBR, TAR) with periodontal status—Plaque Index (PI), Gingival Index (GI), and the simplified Basic Periodontal Examination (s-BPE; pediatric index)—within the T1DM cohort, adjusting for key sociodemographic and oral-hygiene covariates. The secondary aim was to compare periodontal outcomes between youth with T1DM and healthy peers. Caries experience (dmft/DMFT) was recorded solely as an exploratory descriptor of overall oral health and was not treated as a periodontal endpoint. We hypothesized that participants with T1DM would show higher PI, GI, and s-BPE than controls, and that adverse glycemic profiles (higher CV and TBR/TAR, lower TIR) would be independently associated with worse periodontal indices.

## Methods

### Study design and oversight

This single-center, comparative cross-sectional study evaluated oral health in children with T1DM and examined associations between glycemic control parameters (HbA1c and continuous glucose monitoring [CGM] metrics) and periodontal outcomes. The study was jointly conducted by the Department of Pediatric Endocrinology (Muğla Sıtkı Koçman University Faculty of Medicine) and the Department of Pediatric Dentistry (Faculty of Dentistry). Ethical approval was obtained from the Muğla Sıtkı Koçman University Institutional Ethics Committee (28 Oct 2024; Decision No. 137; Protocol No. 240160). Written informed consent was obtained from parents/legal guardians of participants < 16 years; assent was obtained from children as appropriate. The study adhered to the Declaration of Helsinki and followed STROBE recommendations for cross-sectional studies.

### Sample size

Sample size was calculated using GPower v3.1.9.6 (F tests; ANOVA fixed effects, omnibus; α = 0.05, two-sided; power = 0.95; effect size f = 0.479, based on Ferizi, 2022) [5]. The planned sample size was *N* = 72 (*n* = 24 per group), and the achieved sample comprised *N* = 78 (*n* = 26 per group).

### Participants and recruitment

Seventy-eight volunteers aged 7–12 years were allocated to three groups: T1DM with HbA1c < 7% (*n* = 26), T1DM with HbA1c ≥ 7% (*n* = 26), and healthy controls (*n* = 26). Sex was balanced in each group (13 male, 13 female). Children with diabetes were recruited from the pediatric endocrinology outpatient clinic; healthy controls were recruited during routine pediatric dentistry check-ups.

Controls were screened using a structured, parent-completed medical history and review of systems; eligible controls had no systemic disease and no symptoms suggestive of diabetes. Height and weight were measured and body mass index (BMI) converted to age-/sex-specific z-scores; per eligibility, controls were non-obese (BMI < 95th percentile) [29]. For ethical reasons, venipuncture was not performed in controls; therefore, HbA1c/CGM metrics were unavailable for the healthy group. Accordingly, HbA1c/CGM analyses were confined to the T1DM cohort, whereas oral health outcomes were compared across the three groups.

### Eligibility criteria

For the T1DM (case) group, participants were children aged 7–12 years with a ≥ 1-year history of type 1 diabetes mellitus, active CGM use providing ≥ 14 days of data within the 30 days preceding the dental examination (real-time or intermittently scanned CGM), no periodontal treatment within the past 6 months, and no systemic disease other than T1DM. Healthy controls were systemically healthy, age- and sex-comparable to cases (7–12 years), non-obese (BMI < 95th percentile for age/sex), and had no periodontal treatment within the past 6 months. Exclusion criteria (applied as specified) were: age < 7 or > 12 years; chronic medication use likely to affect periodontal tissues; professional periodontal treatment within the past 6 months; current orthodontic treatment; and active infection (e.g., pharyngitis, tonsillitis, rhinitis). For the case group specifically, additional exclusions were systemic disease other than T1DM and T1DM duration < 1 year.

### Glycemic assessment (T1DM only)

Glycemic control was characterized using the most recent clinic HbA1c and CGM summaries from the preceding 14–30 days: coefficient of variation (CV, %), time in range (TIR, 70–180 mg/dL), time below range (TBR, < 70 and < 54 mg/dL), and time above range (TAR, > 180 and > 250 mg/dL), per pediatric consensus guidance. Low GV was defined as CV < 36% and high GV as CV ≥ 36% [13].

### Questionnaire and covariates

Before the dental examination, parents completed a study-specific questionnaire (Supplementary File 1) capturing the child’s age, sex, and—if applicable—age at T1DM diagnosis; parental age, education, occupation; household income; place of residence (urban/rural); and oral-hygiene behaviors: tooth-brushing frequency, use of dental floss and/or mouthwash (yes/no), xerostomia (yes/no), and routine dental visits (6-monthly, yearly, or as needed). In healthy controls, the structured history was also used to exclude systemic disease. Dietary frequency of free-sugar intake was not collected.

### Oral examination and indices

A single calibrated pediatric dentist conducted all examinations under standardized operatory light using a WHO ball-ended periodontal probe (0.5-mm tip, color-coded bands) [30]. Before data collection, the examiner completed procedural calibration on 10 non-study children using the study indices—Plaque Index (PI), Gingival Index (GI), and the pediatric simplified Basic Periodontal Examination (s-BPE)—under identical conditions [31–33]. Calibration focused on consistent probing force and scoring interpretation through a structured review of index criteria, consensus rating of exemplar cases, and use of a written scoring manual/standard operating procedure. All study examinations were performed by this examiner to minimize measurement variability. Formal inter- or intra-examiner reliability coefficients (e.g., κ/ICC) were not calculated; therefore, calibration is reported qualitatively and acknowledged as a limitation.

Two plaque–gingival indices were recorded (0–3). PI reflected plaque accumulation as originally defined (0 = none; 1 = detectable only by probe; 2 = visible/moderate; 3 = abundant). GI reflected inflammation severity with bleeding criteria (0 = normal; 1 = mild, no bleeding on probing; 2 = moderate, bleeding on probing; 3 = severe, spontaneous bleeding/ulceration) [31, 32]. Both primary and permanent teeth present were examined. For PI and GI, six surfaces per tooth (mesiobuccal, mid-buccal, distobuccal, mesiolingual/palatal, mid-lingual/palatal, distolingual/palatal) were scored; the tooth-level value was the mean of its six surfaces, and the participant-level PI/GI was the mean across all examined teeth. No plaque-disclosing solution was used.

The s-BPE followed the joint BSP/BSPD pediatric guideline [33]. Six index teeth (16, 11, 26, 36, 31, 46) were examined with a WHO ball-ended periodontal probe (0.5-mm tip). Codes 0–2 were used for children aged 7–11 years, whereas the full range (0, 1, 2, 3, 4, *) was applied at age 12. Participant-level s-BPE was summarised as the highest code observed across the six index teeth.

Dental caries was recorded per WHO criteria using dmft (primary) and DMFT (permanent). For both dentitions, decayed, missing due to caries, and filled teeth were counted; unerupted and physiologically exfoliated primary teeth were excluded. Only cavitated lesions were scored as “decayed.” dmft and DMFT were calculated separately and reported as exploratory descriptors of overall oral health, in addition to periodontal indices. No additional invasive procedures or diagnostic tests were performed during oral assessments; all data were obtained from routine clinical evaluations and existing records [30].

### Statistical analysis

Data were analyzed using IBM SPSS Statistics version 30 (IBM Corp., Armonk, NY, USA). Descriptive statistics were presented as number (n), percentage (%), mean ± standard deviation, median, and interquartile range (IQR). Normality was assessed with the Shapiro–Wilk test and homogeneity of variances with Levene’s test. For two-group comparisons, independent-samples t-tests or Mann–Whitney U tests were used as appropriate. For comparisons across three or more groups, Kruskal–Wallis tests with Dunn–Bonferroni post-hoc adjustments were applied. Associations between numerical variables were evaluated using Spearman’s rank correlation, and agreement between HbA1c (< 7% vs. ≥ 7%) and CV (< 36% vs. ≥ 36%) classifications was assessed using Cohen’s kappa. Categorical variables were compared using Pearson’s chi-square, Fisher–Freeman–Halton, or Fisher’s exact tests (with Yates’ continuity correction when appropriate).

Because HbA1c/CGM metrics were not available in healthy controls, multivariable analyses were restricted to the T1DM cohort. To identify factors associated with periodontal health parameters (plaque index, gingival index), we fitted multiple linear regression models using backward elimination to obtain the final model, entering HbA1c and CGM-derived metrics (CV, TIR, TBR < 70 mg/dL, TBR < 54 mg/dL, TAR > 180 mg/dL, TAR > 250 mg/dL) as candidate predictors. Models were adjusted a priori for age, sex, maternal and paternal ages, parental education levels, and household income, with categorical predictors entered as dummy variables. Model assumptions (linearity, normality and homoscedasticity of residuals, and multicollinearity) were checked. Two-sided p-values < 0.05 were considered statistically significant.

## Results

We analyzed 78 participants (T1DM: *n* = 52; healthy controls: *n* = 26). Sex distribution was balanced (50% female) and did not differ by group (*p* = 1.000). There were no significant differences between diabetic and control groups in age, maternal age, or paternal age (*p* > 0.05). Parental education and household income were also comparable (*p* > 0.05). Oral-hygiene behaviors (toothbrushing frequency, use of dental floss, routine dental visits) did not differ across groups (*p* > 0.05); no participant reported antimicrobial mouthrinse use. Xerostomia was reported only in the T1DM group (30.8%) and differed significantly from controls (*p* = 0.004) (Table 1).

**Table 1:** Participant characteristics by group (T1DM vs. controls)

	Groups		Test Statistics	
	Controls *n* = 26	T1DM *n* = 52	Test Value	*p* value
Gender, *n* (%)				
Female	13 (50.0)	26 (50.0)	-	-
Male	13 (50.0)	26 (50.0)		
Age, (*years*)	10.2 ± 1.5	10.3 ± 1.3	0.296	0.768^†^
BMI SDS (*mean ± SD)*	0.28 (1.31)	0.05 (1.74)	1.314	0.189^†^
Maternal Age, (*years*)	40.4 ± 5.7	39.4 ± 5.9	0.755	0.452^†^
Paternal Age, (*years*)	45.8 ± 6.0	43.8 ± 5.7	1.374	0.173^†^
Maternal education level, *n* (%)				
Primary school	10 (38.5)	15 (28.8)		
Middle school	7 (26.9)	14 (26.9)	0.893	0.827^‡^
High school	5 (19.2)	13 (25.0)		
University	4 (15.4)	10 (19.2)		
Paternal education level, *n* (%)				
Primary school	2 (8.3)	17 (33.4)		
Middle school	6 (25.0)	7 (13.7)	7.167	0.067^‡^
High school	6 (25.0)	15 (29.4)		
University	10 (41.7)	12 (23.5)		
Household income level, *n* (%)				
Low	2 (7.7)	3 (5.8)		
Middle	10 (38.5)	23 (44.2)	0.465	0.869^¥^
High	14 (53.8)	26 (50.0)		
Tooth brushing frequency, *n* (%)				
Twice a day	13 (50.0)	17 (32.7)		
Once a day	10 (38.5)	26 (50.0)	2.225	0.329^‡^
Occasionally	3 (11.5)	9 (17.3)		
Use of dental floss, *n* (%)				
Yes	0 (0.0)	1 (1.9)	-	1.000^ŧ^
No	26 (100.0)	51 (98.1)		
Use of antimicrobial agents, *n* (%)	0 (0.0)	0 (0.0)	-	NA
Presence of dry mouth, *n* (%)				
Yes	0 (0.0)	16 (30.8)	8.266	0.004 ^Փ^
No	26 (100.0)	36 (69.2)		
Frequency of dental visits, *n* (%)				
Every 6 months	1 (3.8)	2 (3.8)		
Once a year	4 (15.4)	10 (19.2)	0.348	0.892^¥^
When needed	21 (80.8)	40 (76.9)		

*T1DM* Type 1 Diabetes mellitus *n* Number of participants, *%* Column percentage, numerical variables are presented as mean ± standard deviation

†: Independent samples t test, ‡: Pearson chi-square test, ¥: Fisher-Freeman-Halton exact test, Փ: Yates’ chi-square test, ŧ: Fisher’s exact test

Within the T1DM cohort (controls had no HbA1c/CGM data), agreement between the binary classifications—HbA1c ≥ 7% vs. < 7% and GV (CV ≥ 36% vs. < 36%)—was 80.8% (Cohen’s κ = 0.615, *p* < 0.001), indicating substantial agreement (Table 2).

**Table 2:** Agreement between HbA1c category (< 7% vs. ≥ 7%) and GV category (CV < 36% vs. ≥ 36%) in the T1DM cohort (*n* = 52)

		CV		Total	Agreement Statistics	
		< 36%	≥ 36%		Kappa	*p-value*
HbA1c	< 7%	21	5	26	0.615	< 0.001
	≥ 7%	5	21	26		
Total		26	26	52		

Cell values are *n*; totals are row/column sums. Agreement assessed with Cohen’s κ. Percent agreement: 80.8% (42/52)

*HbA1c * hemoglobin A1c, *CV* coefficient of variation, *GV* glycemic variability

For descriptive group contrasts, we present three clinical groups (T1DM with HbA1c < 7%, T1DM with HbA1c ≥ 7%, and healthy controls); note that HbA1c/GV were not measured in controls, so etiologic inferences from HbA1c/GV are drawn within T1DM below. Group differences in PI, GI and s-BPE were tested with Kruskal–Wallis, and Bonferroni-adjusted Dunn–Bonferroni post hoc tests were used for pairwise contrasts.

DMFT/dmft did not differ significantly across the three groups (*p* > 0.05). In contrast, PI, GI, and s-BPE were higher in diabetic participants than in healthy controls (*p* = 0.001, *p* < 0.001, *p* < 0.001, respectively). No differences were observed between the two T1DM HbA1c groups for these periodontal parameters (Table 3).

**Table 3:** Dental outcomes by HbA1c-based glycemic control (HbA1c < 7% vs. ≥ 7%) and healthy controls

	Groups			Test Statistics	
	HbA1c < 7% *n* = 26	HbA1c ≥ 7% *n* = 26	Controls *n* = 26	Test value	*p* value
dmft (primary teeth)	1.00 (4.00)	0.00 (4.00)	0.00 (4.00)	0.444	0.801^§^
DMFT (permanent teeth)	0.50 (2.25)	1.00 (2.25)	2.00 (3.25)	4.908	0.086^§^
Plaque index	1.23 (0.69) ^*a*^	1.19 (0.53) ^*a*^	0.39 (0.50) ^*b*^	29.562	< 0.001 ^§^
Gingival index	1.00 (0.07) ^*a*^	1.00 (0.10) ^*a*^	0.11 (0.58) ^*b*^	28.637	< 0.001 ^§^
s-BPE	1.04 (0.37) ^*a*^	1.08 (0.58) ^*a*^	0.50 (0.27) ^*b*^	16.101	< 0.001 ^§^

Data are median (IQR). § Kruskal–Wallis H(2) across three groups (HbA1c < 7%, HbA1c ≥ 7%, controls); Dunn–Bonferroni–adjusted post-hoc pairwise tests. Superscript letters (a, b) indicate pairwise differences; groups sharing a letter do not differ

Controls were included as an external comparator; HbA1c was not measured in controls

*Abbreviations*: *HbA1c* glycated hemoglobin, *PI* Plaque Index, *GI* Gingival Index, *s-BPE* simplified Basic Periodontal Examination, *dmft/DMFT* decayed–missing–filled teeth (primary/permanent).

Considering T1DM low GV (CV < 36%), T1DM high GV (CV ≥ 36%), and healthy controls (controls included only as an external comparator), permanent-tooth DMFT differed across groups (Kruskal–Wallis *p* = 0.045); the low-GV vs. healthy pair remained significant after Bonferroni correction, while other pairwise differences were not significant. Similarly, diabetic participants (both GV strata) had higher PI, GI, and s-BPE than controls (*p* < 0.001 for all), with no difference between low- and high-GV T1DM groups (Table 4).

**Table 4:** Comparison of dental variables according to CV-Based glycemic control classification

	Groups			Test Statistics	
	CV < 36% *n* = 26	CV ≥ 36% *n* = 26	Controls *n* = 26	Test value	*p* value
dmft (primary teeth)	1.00 (4.00)	0.00 (3.50)	0.00 (4.00)	0.533	0.766^§^
DMFT (permanent teeth)	0.00 (2.00) ^*a*^	1.00 (3.00) ^*ab*^	2.00 (3.25) ^*b*^	6.185	0.045 ^§^
Plaque index	1.10 (0.53) ^*a*^	1.42 (0.78) ^*a*^	0.39 (0.50) ^*b*^	29.926	< 0.001 ^§^
Gingival index	1.00 (0.03) ^*a*^	1.02 (0.43) ^*a*^	0.11 (0.58) ^*b*^	30.810	< 0.001 ^§^
s-BPE	1.00 (0.36) ^*a*^	1.25 (0.75) ^*a*^	0.50 (0.27) ^*b*^	17.483	< 0.001 ^§^

Data are median (IQR). § Kruskal–Wallis H(2) across three groups (CV < 36%, CV ≥ 36%, controls); Dunn–Bonferroni–adjusted post-hoc pairwise tests. Superscript letters (a, b) indicate pairwise differences; groups sharing a letter do not differ

*Abbreviations:* *PI* Plaque Index, *GI* Gingival Index, *s-BPE* simplified Basic Periodontal Examination, *dmft/DMFT* decayed–missing–filled teeth (primary/permanent)

Sex and parental age were not associated with dental outcomes (*p* > 0.05). Age showed a moderate negative correlation with dmft (*r* = − 0.435, *p* < 0.001). Maternal education was associated with DMFT (*p* < 0.05), with lower DMFT in children of mothers with high-school/university education. No differences were observed in other dental parameters by maternal education; paternal education and household income were not associated with any oral-health outcome. Urban vs. rural residence showed no differences (*p* > 0.05).

Among glycemic parameters, time-below-range < 70 mg/dL (TBR < 70) was weakly and positively correlated with PI (Spearman ρ = 0.312, *p* < 0.05) and GI (ρ = 0.354, *p* < 0.05). No other significant associations were observed between glycemic metrics (including HbA1c, CV, TIR, TAR) and oral-health indicators (Table 5).

**Table 5:** Spearman correlations between glycemic metrics (HbA1c and CGM-derived variables) and dental outcomes in the T1DM cohort (*n* = 52)

	dmft (primary teeth)	DMFT (permanent teeth)	Plaque index	Gingival index	s-BPE
HbA1c	−0.099; 0.484	0.150; 0.290	−0.002; 0.989	0.066; 0.640	0.162; 0.257
CV	0.040; 0.780	0.164; 0.245	0.151; 0.285	0.219; 0.119	0.178; 0.211
TIR	0.113; 0.426	−0.141; 0.317	0.054; 0.703	−0.036; 0.801	−0.082; 0.567
TBR70	0.094; 0.506	0.269; 0.054	**0.312; 0.024**	**0.316; 0.022**	0.230; 0.104
TBR54	0.159; 0.261	0.267; 0.056	0.248; 0.077	0.266; 0.057	0.255; 0.071
TAR180	−0.087; 0.539	0.123; 0.385	−0.151; 0.284	−0.115; 0.416	0.030; 0.837
TAR250	0.003; 0.984	0.051; 0.720	−0.095; 0.503	0.015; 0.918	−0.010; 0.945

Values are Spearman’s ρ; *p*-value. Analyses restricted to the T1DM cohort (n = 52); CGM summaries reflect the most recent 14–30 days. TIR = 70–180 mg/dL; TBR = < 70 or < 54 mg/dL; TAR = > 180 or > 250 mg/dL; s-BPE = Simplified Basic Periodontal Examination. CV: Coefficient of Variation, a measure of glycemic variability calculated from continuous glucose monitoring data. HbA1c: Hemoglobin A1c, an indicator of average blood glucose levels over the past 2–3 months

In multivariable linear regression adjusted for demographic covariates, TBR < 54 remained positively associated with PI (β = 0.195; SE = 0.087; 95% CI 0.019–0.370; *p* = 0.030). For GI, TIR (β = 0.013; SE = 0.006; 95% CI 0.001–0.024; *p* = 0.030) and CV (β = 0.037; SE = 0.014; 95% CI 0.008–0.066; *p* = 0.013) showed small-magnitude, directionally unexpected associations. No predictors were significant for s-BPE. Model summaries are shown in Table 6.

**Table 6:** Evaluation of factors associated with periodontal health parameters using multiple linear regression analysis

	Regression Coefficients*						
	*β*	*se*	*zβ*	*t*	*p*	95.0% Confidence Interval for *β*	
						*Lower Bound*	*Upper Bound*
Model-1(Dependent variable: Plaque index)							
TBR54	0.195	0.087	0.316	2.248	0.030	0.019	0.370
Model Summary: *F*=2.069; *p*=0.041; *R*^2^=0.414; *Adj R*^2^=0.214							
Model-2(Dependent variable: Gingival index)							
TIR	0.013	0.006	0.480	2.259	0.030	0.001	0.024
CV	0.037	0.014	0.508	2.614	0.013	0.008	0.066
Model Summary: *F*=2.147; *p*=0.032; *R*^2^=0.448; *Adj R*^2^=0.240							
Model-3(Dependent variable: s-BPE)							
No glycemic predictors retained							

For all models

Adjusted for sex, age, maternal age, paternal age, maternal education level, paternal education level, household income levelPredictors entered on step 1: Hba1c, CV, TIR, TBR70, TBR54, TAR180, TAR250

Analyses restricted to the T1DM cohort (n=52); CGM summaries reflect the most recent 14–30 days. TIR = 70–180 mg/dL; TBR = <70 or <54 mg/dL; TAR = >180 or >250 mg/dL; s-BPE = Simplified Basic Periodontal Examination. CV: Coefficient of Variation, a measure of glycemic variability calculated from continuous glucose monitoring data. HbA1c: Hemoglobin A1c, an indicator of average blood glucose levels over the past 2–3 months

## Discussion

In this comparative cross-sectional study of children with T1DM, periodontal status was worse than in healthy peers, with higher plaque index (PI), gingival index (GI), and simplified Basic Periodontal Examination (s-BPE) scores. Subgroup analyses by HbA1c and by glycemic variability (coefficient of variation, CV) showed no between-group differences in periodontal indices. In exploratory analyses, time below range (TBR, < 70 mg/dL) exhibited a weak positive association with PI and GI. Self-reported xerostomia was more frequent in T1DM. Regarding caries, maternal education was inversely associated with DMFT, whereas periodontal outcomes did not differ between rural and urban residents in our cohort.

Our finding of higher plaque/gingival inflammation and worse s-BPE in T1DM is consistent with pediatric evidence. Comparative cross-sectional and case–control studies show higher plaque, gingival index, bleeding-on-probing, and a greater proportion of sites with PD > 3 mm in youth with T1DM versus healthy peers [3, 18]. Some cohort report plaque increases without significant differences in gingival indices or bleeding [34]. Large pediatric analyses also indicate higher odds of periodontal destruction even at young ages [8]. Microbiome-oriented pediatric studies suggest a shift toward cariogenic/periodontopathogenic profiles with poorer metabolic control—e.g., higher Streptococcus mutans (and, in some cohorts, co-carriage with Veillonella)—which aligns with greater plaque accumulation and gingival inflammation [4, 35, 36].

Despite the overall periodontal gap between groups, we did not detect significant differences when T1DM participants were stratified by HbA1c or by glycemic variability (CV), in line with pediatric data showing no periodontal separation between well- and poorly controlled subgroups [3]. Conversely, other pediatric work reports continuous associations—higher HbA1c tracking with higher PI, GI, BOP and more sites with PD > 3 mm [11]. Heterogeneity is also evident across pediatric cross-sectional datasets, where gingival inflammation may differ while plaque accumulation does not [37]. A recent meta-analysis likewise found pooled plaque/gingival differences to be often non-significant, while differences in caries experience and salivary flow were clearer, particularly under poor glycemic control [38]. Methodologically, dichotomizing glycemia can mask effects, and HbA1c and CGM capture different time windows (≈ 0.8% HbA1c per ~ 10% change in TIR); thus, null HbA1c-strata contrasts do not preclude time-based glycemic effects [15].

Consistent with our primary hypothesis, greater hypoglycemia exposure (TBR < 54 mg/dL) was associated with higher plaque levels after adjustment. By contrast, other CGM metrics exhibited only small and directionally inconsistent links (TIR and CV with the gingival index), which are unlikely to reflect true biological effects and therefore warrant cautious interpretation (Table 6). Overall, these patterns support focusing on hypoglycemia-related behaviors as a modifiable conduit while acknowledging that cross-sectional CGM–periodontal associations may be subtle and context-dependent.

Building on this signal, the weak positive correlation between TBR < 70 mg/dL and PI/GI is, to our knowledge, among the first pediatric observations linking hypoglycemia exposure to periodontal status. Pediatric guidelines define < 70 mg/dL as an alert threshold and recommend TBR < 4% (and < 1% for < 54 mg/dL) [13]. Clinically, recurrent hypoglycemia—especially at night—often entails rapid-acting sugar intake; if post-event hygiene is skipped, frequent free-sugar exposures can plausibly increase plaque and gingival inflammation. This interpretation coheres with WHO guidance to limit free sugars to < 10% of energy (preferably < 5%) to reduce caries risk and supports a common-risk-factor counseling approach within diabetes care [24]. Importantly, randomized dietary trials show that anti-inflammatory dietary patterns can reduce gingival inflammation even when plaque levels do not change, underscoring a host-modulation pathway that could amplify the behavioral link between hypoglycemia management and gingival outcomes [25, 26]. Our exploratory finding therefore warrants confirmation in prospective designs with detailed nocturnal behavior logging and CGM phenotyping.

The higher frequency of xerostomia in T1DM observed here is consistent with evidence of reduced resting/stimulated salivary flow and compositional changes in diabetes—mechanistically linked to hyperglycemia, microvascular involvement, and autonomic neuropathy [39, 40]. Pediatric datasets likewise indicate lower salivary flow and buffering capacity and higher cariogenic/acidogenic counts in children with T1DM and/or under poorer metabolic control [5, 6, 18]. Such alterations plausibly facilitate plaque accumulation and modulate periodontal inflammatory responses, dovetailing with our periodontal findings.

Recognizing that dmft/DMFT quantify caries burden rather than periodontal inflammation, we analyze caries outcomes separately from periodontal indices. Across studies, caries tend to be higher under poor metabolic control, whereas in some settings caries are similar between children with T1DM and controls or even higher among controls [5, 18, 41–43]. Meta-analytic data indicate that caries prevalence in children with T1DM remains high (~ 67%) and is lower with better metabolic control [38, 44]. This heterogeneity likely reflects differences in hygiene routines, diet, and care patterns, alongside diabetes-related salivary changes that can elevate caries risk [6, 38]. In our cohort, the inverse association between maternal education and DMFT aligns with this context-sensitive pattern and underscores the need to tailor caries prevention to behavioral and salivary milieus, distinct from periodontal inflammation.

Our cohort’s finding that higher maternal education associates with lower caries aligns with evidence that parental education and oral-health literacy shape children’s oral-health behaviors and status—caregiver literacy correlates with clinically assessed disease severity and with parent-reported status, and lower literacy is linked to deleterious behaviors such as night-time bottle use and less daily brushing [22, 45]. In pediatric T1DM populations, higher parental education predicts better oral-health knowledge, attitudes, and practices [46]. Turkish data link greater parental health literacy with better glycemic outcomes in younger children—suggesting an indirect pathway to oral outcomes [47]. Consistent with this, a systematic review shows that lower parental socioeconomic status, passive smoking exposure, and a parental history of periodontal disease are associated with poorer periodontal status in youth, underscoring the household as a key locus for intervention alongside metabolic targets [21].

We found no rural–urban differences in periodontal indicators, which may reflect our province’s high socioeconomic development level and relatively equitable access to preventive and restorative oral-health services [48]. Nonetheless, populations with restricted access—often in lower socioeconomic status (SES) or rural settings—experience worse oral outcomes driven by social and commercial determinants (including free-sugar availability and marketing) [20]. Larger multi-site studies spanning diverse development indices are needed to clarify geography-by-context effects.

Our findings support integrating oral health into pediatric diabetes services as part of SDG-3’s noncommunicable disease prevention and universal health coverage agenda [49]. Oral diseases share modifiable risk factors with diabetes—particularly free sugars and social/behavioral determinants—so a common-risk-factor approach can deliver co-benefits [20, 23, 24]. Embedding hypoglycemia-management education with night-time oral hygiene and free-sugar minimization (e.g., choosing lower-sugar options and prompt toothbrushing/mouth-rinsing after treatment) operationalizes this framework for families [23, 24]. At the system level, strengthening primary-care linkages between pediatric endocrinology and dentistry, improving parental/oral-health literacy, and targeting high-risk settings can reduce inequities highlighted by global oral-health analyses [20, 22, 45, 46]. Given the elevated long-term periodontal risk in T1DM populations, integrated, prevention-first models are well-positioned to be implemented within routine diabetes follow-up and directly advance SDG-3 targets on NCDs and UHC [50].

A key strength of this study is the comprehensive characterization of glycemia beyond HbA1c, incorporating consensus continuous-glucose-monitoring metrics—glycemic variability (CV), time in range (TIR), and time below range (TBR)—which enables a more nuanced appraisal of periodontal associations in youth. The use of pediatric-appropriate, standardized periodontal indices (PI, GI, s-BPE) under uniform examination conditions further enhances measurement rigor. All examinations were performed by a single, calibrated examiner using a WHO ball-ended periodontal probe with prespecified scoring procedures, and inclusion of a healthy control group strengthens the comparative interpretation of findings.

The cross-sectional, single-center design precludes causal inference and may limit generalizability. Subgroup contrasts had modest statistical power, warranting cautious interpretation. Examiner calibration was qualitative and formal intra-examiner reliability coefficients (e.g., κ/ICC) were not obtained; examiner blinding to group was not feasible. HbA1c/CGM data were unavailable in controls, restricting multivariable modeling to the T1DM cohort. Behavioral covariates relied on self-report, and potentially relevant factors—such as the frequency of dietary free-sugar exposures and the use of plaque-disclosing agents—were not measured. Taken together, these limitations support cautious interpretation and underscore the need for prospective, multicenter studies with larger samples, objective behavioral assessments, and broadened oral-health phenotyping (e.g., salivary function) to clarify mechanistic pathways.

In conclusion, children with T1DM exhibited worse periodontal status than healthy peers. Our exploratory observation that greater hypoglycemia exposure (higher TBR) coincided with higher plaque and gingival indices suggests a modifiable behavioral conduit—hypoglycemia-management practices—operating alongside salivary dysfunction and social determinants. Embedding CGM-informed glycemic targets with behavior-specific oral-health counseling (e.g., free-sugar guidance and night-time hygiene) is well-positioned to be implemented within pediatric diabetes care and aligns with SDG-3-oriented prevention.

## Supplementary Information

Supplementary Material 1

## Data Availability

The datasets generated and/or analyzed during the current study are available from the corresponding author on reasonable request.
