# Supplementary material for: Association of glycemic variability and sociodemographic determinants with periodontal health in children with type 1 diabetes: a cross-sectional study from Türkiye
Source: BMC Oral Health. 2026 Feb 28;26:401. doi: 10.1186/s12903-025-07484-z (PMC12950240; doi:10.1186/s12903-025-07484-z)
Supplement: Supplementary file 1 — Supplementary Material 1 [file 12903_2025_7484_MOESM1_ESM.docx]

**Supplementary File 1. Structured Questionnaire Used for Data Collection**

**Section 1. Demographic Information**

**Child’s Age:** .....................

**Gender:** Male ( ) Female ( )

**Age at Diagnosis of Type 1 Diabetes:** .....................

**Mother’s Age:** .....................

**Father’s Age:** .....................

**Mother’s Education Level:**

( ) Primary school ( ) Middle school ( ) High school ( ) University

**Father’s Education Level:**

( ) Primary school ( ) Middle school ( ) High school ( ) University

**Section 2. Socioeconomic Status**

**Family income level:**

( ) Low (below the national minimum wage in Türkiye, <17,000 TRY/month)

( ) Middle (between 17,000 and 34,000 TRY/month; approximately 1–2× minimum wage)

( ) High (above 34,000 TRY/month; more than 2× minimum wage)

**Section 3. Medical and Oral Health Information**

**Tooth Brushing Habit:**

( ) Twice a day ( ) Once a day ( ) Occasionally

**Use of Dental Floss:**

( ) Yes ( ) No

**Use of Antimicrobial Agents** **(e.g., mouthwash with chlorhexidine):**

( ) Yes ( ) No

**Presence of Dry Mouth Sensation:**

( ) Yes ( ) No

**Frequency of Dental Visits:**

( ) Every 6 months ( ) Once a year ( ) When needed

*Note: This questionnaire was developed by the authors specifically for this study and was used to collect sociodemographic and oral health-related data from children with Type 1 Diabetes and their caregivers.*
